# Pilot Study on Resuscitation Volume’s Effect on Perfusion and Inflammatory Cytokine Expression in Peri-Burn Skin: Implications for Burn Conversion

**DOI:** 10.3390/ebj6030042

**Published:** 2025-07-28

**Authors:** Tamer R. Hage, Edward J. Kelly, Eriks Ziedins, Babita Parajuli, Cameron S. D’Orio, David M. Burmeister, Lauren Moffatt, Jeffrey W. Shupp, Bonnie C. Carney

**Affiliations:** 1 Firefighter’s Burn and Surgical Research Laboratory, MedStar Health Research Institute, Washington, DC 20010, USA; tamer.hage@medstar.net (T.R.H.); kelly.ted@gmail.com (E.J.K.); eriks.ziedins@gmail.com (E.Z.); camerondorio@gmail.com (C.S.D.); lauren.t.moffatt@medstar.net (L.M.); jeffrey.w.shupp@medstar.net (J.W.S.); 2 Departments of Medicine and Surgery, Uniformed Services University of the Health Sciences, Bethesda, MD 20814, USA; babita.parajuli.ctr@usuhs.edu (B.P.); david.burmeister@usuhs.edu (D.M.B.); 3 Departments of Biochemistry and Surgery, Georgetown University School of Medicine, Washington, DC 20007, USA; 4 The Burn Center, Department of Surgery, MedStar Washington Hospital Center, Washington, DC 20010, USA; 5 Department of Plastic Surgery, Georgetown University School of Medicine, Washington, DC 20007, USA

**Keywords:** burn wound progression (BWP), skin inflammation, laser doppler imaging (LDI), fluid resuscitation, terminal deoxynucleotidyl transferase dUTP nick end labeling (TUNEL)

## Abstract

Fluid resuscitation after thermal injury is paramount to avoid burn shock and restore organ perfusion. Both over- and under-resuscitation can lead to unintended consequences affecting patient outcomes. While many studies have examined systemic effects, limited data exist on how fluid resuscitation impacts burn wound progression in the acute period. Furthermore, the mechanisms underlying burn wound progression remain not fully understood. This study used a swine model to investigate how varying resuscitation levels affect peri-burn wound dynamics. Twenty-seven female Yorkshire pigs were anesthetized, subjected to 40% total body surface area burn and 15% hemorrhage, then randomized (*n* = 9) to receive decision-support-driven (adequate, 2–4 mL/kg/%TBSA), fluid-withholding (under, <1 mL/kg/%TBSA), or high-constant-rate (over, >>4 mL/kg/%TBSA) resuscitation. Pigs were monitored for 24 h in an intensive care setting prior to necropsy. Laser Doppler Imaging (LDI) was conducted pre-burn and at 2, 6, 12, and 24 h post burn to assess perfusion. Biopsies were taken from burn, peri-burn (within 2 cm), and normal skin. RNA was isolated at 24 h for the qRT-PCR analysis of IL-6, CXCL8, and IFN-γ. At hour 2, LDI revealed increased peri-burn perfusion in over-resuscitated animals vs. under-resuscitated animals (*p* = 0.0499). At hour 24, IL-6 (*p* = 0.0220) and IFN-γ (*p* = 0.0253) were elevated in over-resuscitated peri-burn skin. CXCL8 showed no significant change. TUNEL staining revealed increased apoptosis in over- and under-resuscitated peri-burn skin. Differences in perfusion and cytokine expression based on resuscitation strategy suggest that fluid levels may influence burn wound progression.

## 1. Introduction

Thermal burns represent up to 86% of cases of burn injury, disproportionally affect those of lower socioeconomic status, and constitute one of the leading causes of death in children aged 1–4 [1]. In the first two days following a large thermal burn injury, intravascular fluid leaks into tissues and leads to hypovolemia, which, in turn, impairs blood flow and oxygen delivery to the internal organ [2]. This process, named burn shock, can be fatal, and is similar to detrimental processes that can occur after blood loss (hemorrhagic shock), yet it also has components of distributive and cardiogenic shock [3]. Treatment to prevent burn shock and restore hemodynamics and organ perfusion includes the current standard of care: early and aggressive intravenous (IV) fluid resuscitation [4].

Widely practiced in nearly all burn centers is the administration of IV lactated ringers (LRs) titrated to urine output (UO) at 0.5–1.0 cc/kg/h. This variable, used by over 90% of burn centers, is therefore one of the primary metrics currently being used to determine the personalized fluid needs of burn patients [5]. Reliance on this metric has often been criticized as inadequate, and as a result, there is a general lack of agreement among burn professionals as to how to adequately best resuscitate patients in burn shock. This has led to an expanding area of research examining additional parameters that can be used to evaluate adequate resuscitation. For example, utilizing colloids or other blood products (e.g., fresh frozen plasma) to tailor fluid needs is now part of clinical practice in some burn centers and the topic of much research [1,5,6,7,8]. Developing novel criteria capable of determining adequate fluid needs is paramount because under- or over-resuscitation may lead to significant yet avoidable consequences such as “fluid creep,” which exacerbates edema-related tissue damage [9]. Some of these commonly cited edema-related tissue damages include compartment syndrome (including in areas of non-burn limbs) and the swelling of the face and airway, causing complications with airway management, pleural effusions, and respiratory and cardiac failure [9]. Less commonly considered is the tissue-level damage of over-resuscitation at the skin level despite the fact that unburned skin may become the source for autologous skin grafts for wound closure.

**Figure 1:**
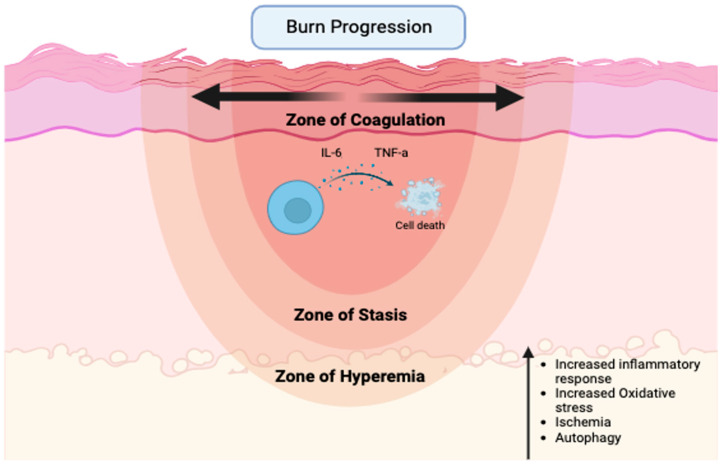
Major factors contributing to the expansion of necrotic tissue following severe thermal injury (created with biorender.com).

Specifically, over-resuscitation may exacerbate burn wound progression, which is the process of partial-thickness burns progressing to non-viable tissue-deep partial-thickness or full-thickness burns following injury [10] (Figure 1).

Peri-burn skin is often referred to as the zone of stasis, an area of tissue directly surrounding the zone of coagulation, which is the site of cell death due to thermal injury. Since the zone of stasis is characterized by ischemia, it is a premier therapeutic target to prevent the loss of perfusion and the subsequent conversion to unrecoverable necrotic tissue that typically occurs over a 12–24 h period. Surrounding the zone of stasis is the zone of hyperemia, which is typically deemed recoverable [9,11]. While many studies are dedicated to the study of the systemic effects of resuscitation, additional local skin tissue-level effects have largely been ignored. Currently, limited information exists on how the quantity of fluid resuscitation affects the burn wound, specifically burn wound progression, in the acute period following shock.

The current understanding of the molecular mechanisms is limited to cytokines [10], reactive oxygen species (ROS), ischemia, and, more recently, autophagy [12]. The most common method for studying burn progression in animals is the “rat comb model” described in a prior review [10]. While the rat comb model has been the golden standard for studying the pathophysiology of burn progression since the 1990s, rodent models do not allow for the implementation of clinical decision support tools or clinically relevant fluid rates and researchers have recently underscored the need to better characterize and standardize the model to reduce variation.

These limitations further complicate the understanding of the mechanisms underlying the phenomena and make the development of therapeutics capable of mitigating the impact of burn progression even more challenging [10,13].

In the current study, we used a large animal model with precisely controlled fluid resuscitation to gain a deeper insight into the mechanisms that promote burn progression in peri-burn skin. We hypothesized that over- and under-resuscitation leads to increased skin inflammation that ultimately leads to ischemia and associated cell death when compared to adequate resuscitation.

## 2. Materials and Methods

### 2.1. Animal Burn Wound Model

Animals were handled according to facility standard operating procedures under the animal care and use program accredited by the Association for Assessment and Accreditation of Laboratory Animal Care International (AAALAC) and Animal Welfare Assurance through the Public Health Service (PHS). All described animal work was reviewed and approved by the MedStar Health Research Institute’s Institutional Animal Care and Use Committee (IACUC) under protocol number MHRI-IACUC 2020-005.

Twenty-seven Yorkshire pigs (average weight: 32.1 kg) were monitored and observed for five or more days prior to experimental use for acclimation in accordance with the George Hyman Research Building Animal Facility requirements. Additional details regarding pre-and post-operative care, anesthesia, and animal monitoring have previously been described by Arabidarrehdor et al. [14].

Baseline full-thickness skin 4 mm punch biopsies were taken from the skin at a site remote to the intended burn injury. A heat source consisting of brass billets was then used to create a 20% total body surface area (TBSA) full-thickness burn on one side. The brass bullets were heated to 150 °C and kept in contact with the skin for 10 s, producing a full-thickness burn as previously described and confirmed by histology [15,16]. Full-thickness skin 4 mm punch biopsies were taken immediately (0–1 h) post burn. Each animal was next turned to its other side and a similar procedure was performed for a total of 40% TBSA burn. The animals were maintained under continuous general anesthesia from injury through the final hour 24 timepoint. The animals were kept warm by utilizing two bair huggers. In addition, all intravenous fluids and drugs were warmed prior to infusion. The animal’s temperature was monitored by esophageal probe and the bair hugger temperatures were titrated to maintain a temperature between 98 and 102 F. The environmental and humidity of the room were not controlled. An arterial line previously inserted for blood collection induced a mild 15% controlled-volume hemorrhage (10–11 mL/kg). The rationale behind inducing a 15% hemorrhage was to limit the ability of the spleen to provide auto-resuscitation to the porcine subject, which would improve their clinical condition even after a 40% burn injury [17,18].

Post burn, pigs were randomized (*n* = 9 in each group) to receive different levels of resuscitation: decision-support-driven (adequate (AR), 2–4 mL/kg/%TBSA), fluid-withholding (under (UR), and only for medications leading to <1 mL/kg/%TBSA) or high constant rates (over (OR), >>4 mL/kg/%TBSA). Groups averaged 0.62 ± 0.13, 3.94 ± 0.37 and 8.63 ± 0.30 mL/kg/%TBSA of fluid respectively.

**Figure 2:**
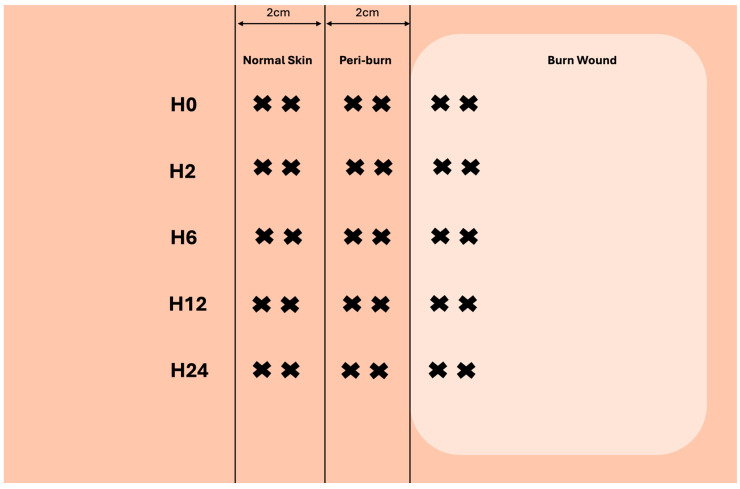
Visualization of skin-punch biopsy collection at each timepoint (created with biorender.com).

Prior to injury, and over the course of 24 h, 4 mm full-thickness biopsies of burned, peri-burn (within 2 cm of burn), and normal skin (>6 cm away from burn) were taken at hours 2, 6, 12, and 24. Figure 2 depicts how multiple biopsies were taken from each site.

### 2.2. Laser Doppler Imaging

Laser Doppler Imaging (LDI) is a noninvasive method of measuring blood flow and burn depth. Mandal et al. have previously defined the physics of LDI, as well as its validity, reproducibility, and reliability [19]. Following burning, LDI was performed using a moorLDI Laser Doppler Line Scanner (Moor Instruments Limited, Axminster, UK) over the burn wound and peri-wound skin pre-burn and at hours 2, 6, 12, and 24 to assess perfusion to burned and peri-burn skin. Statistical analysis was performed using GraphPad Prism () for windows. Mean perfusion was taken from the area adjacent to the site of the direct burn wound (peri-burn skin) at all timepoints, and a two-way ANOVA with Turkey’s multiple comparisons was used to compared resuscitation groups. A *p*-value of <0.05 was considered statistically significant.

### 2.3. Gene Expression Analysis

Skin biopsies were taken from the burn, peri-burn (within 2 cm of the burn), and normal skin areas. RNA was isolated from normal skin prior to injury, and peri-burn skin biopsies at hour 24 using the Rneasy fibrous tissue kit per the manufacturer’s instructions. qRT-PCR was conducted to assess levels of common inflammatory cytokines: interleukin-6 (IL-6), chemokine CXC motif ligand-8 (CXCL8), and interferon-gamma (IFN-y) (Qiagen, Valencia, CA, USA). The ddCt method was used to normalize Ct values to RPL13a as the housekeeper gene and each pig to its own pre-injury level. Data is expressed here as fold change from pre-injury. A >2 or <2-fold change was considered significant.

### 2.4. Terminal Deoxynucleotidyl-Transferase-Mediated Dutp Nick End Labeling (TUNEL) Staining

Skin biopsies taken from the burn, peri-burn, and normal skin prior to burn and at hour 24 were fixed in 10% formaldehyde and embedded in paraffin wax. The skin relevant to this analysis was then sectioned (6 um) and stored for later use.

Peri-burn sections selected from each group (OR, UR, AR) and timepoint (PRE and H24) then underwent TUNEL staining to assess for apoptosis. Staining was performed using reagents from a DeadEnd Colorimetric TUNELtm System kit per the manufacturer’s instructions. In summary, slides containing the sectioned tissue were deparaffinized and rehydrated. The tissue was then fixed with 4% Methanol-Free paraformaldehyde and permeabilized with 20 ug/mL Proteinase K. One slide designated as a positive control next underwent incubation with DNase 1X Buffer solution as well as DNase 10 u/mL. The remaining slides were then protected from light after treatment with a reaction mix containing equilibration buffer, nucleotide mix, and rTDT. One designated negative-control slide was treated with a reaction mix containing equilibration buffer, nucleotide mix, and DI H_2_O instead of rTDT. All the slides next spent 60 min in an incubator at 37 °C covered with aluminum foil. Afterwards, a stop reaction was performed using 2X SCC and the slides were stained with DAPI before being mounted and cover slipped. Immediately after, the slides were imaged using a Zeiss Axioimager under the GFP and DAPI channels using tiled imaging at 40× (Carl Zeiss, Oberkochen, Germany). The slides were next graded as either positive for % rTDT or not compared to group-relevant control slides by an investigator blinded to the study and exposed to both images of the negative- and positive-control slides. The results were evaluated by a Chi-square test.

### 2.5. Statistics

Analysis of gene expression and perfusion units over time was evaluated using a two-way ANOVA and multiple comparisons with Sidak’s correction for multiple comparisons in GraphPad Prism v10.4.1 (GraphPad Software, La Jolla, CA, USA). Values of *p* < 0.05 were considered statistically significant. For all cytokine analyses, ROUT’s outlier test was used to identify outliers. There were no outliers for IL-6, IL-8, or IFN-g. This analysis of cytokine expression in peri-burn skin sites was a sub-analysis of a larger study. The number of animals per group (*n* = 9) was based on a power calculation for the primary outcomes, which was developing an algorithm based on cardiac pressure monitoring. Therefore, these results described here are pilot data for skin cytokine levels.

## 3. Results

### 3.1. Fluid Rates and Urine Outputs Were Different Among Resuscitation Groups

**Figure 3:**
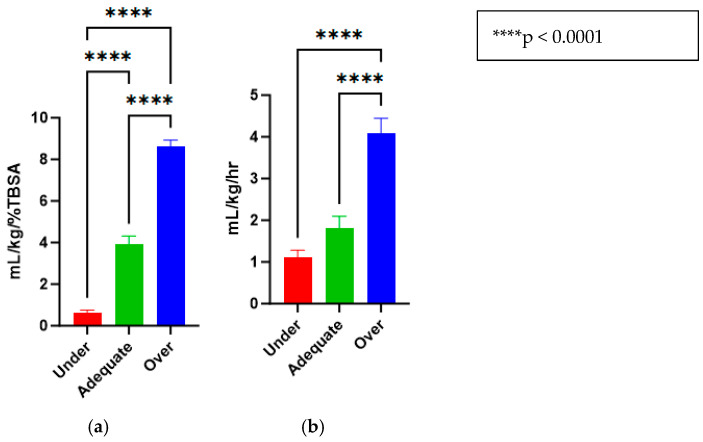
(**a**) Fluid resuscitation rates for under-, adequate-, and over-resuscitation groups, reported in mL/kg/%TBSA. (**b**) Urine outputs for under-, adequate-, and over-resuscitation groups, reported in mL/kg/h.

The treatments for the three groups that were purposefully titrated to receive different fluid levels were successfully accomplished and are reported in mL/kg/%TBSA (Under = 0.62 *±* 0.13, Adequate = 3.94 *±* 0.37, Over = 8.63 *±* 0.30) (Figure 3a). Similarly, the animals had urine outputs in line with what would be expected by delivering these fluid amounts (Under = 1.12 ± 0.16, Adequate = 1.82 *±* 0.27, Over = 4.09 *±* 0.35) (Figure 3b).

### 3.2. Resuscitation Levels Affect Perfusion in Peri-Burn Skin

**Figure 4:**
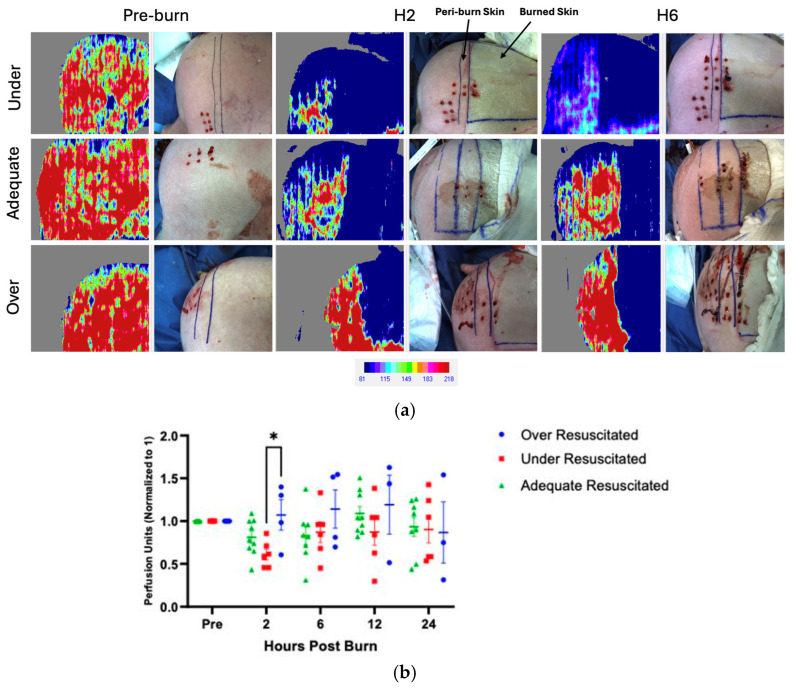
(**a**) LDI at hours 2 and 6 visualized increased perfusion to the peri-burn site of over-resuscitated animals at H2. Each row represents the same individual pig from the under-, adequate-, and over-resuscitation groups shown across three timepoints (Pre-burn, Hour 2, Hour 6) to depict the intraindividual time course. (**b**) LDI analysis demonstrates increased perfusion to the peri-burn site of over-resuscitated animals at H2.

There were varying levels of perfusion in unburned skin pre-injury, and therefore, all data was normalized to each pig’s individual pre-burn uninjured skin level. At hour 2, there was increased perfusion in the peri-burn skin of over-resuscitated animals when compared to under-resuscitated groups (*p* = 0.0499), with no difference by the end of the experiment (Figure 4a,b). There were no significant changes from hours 6 through 24.

### 3.3. Resuscitation Levels Affect Cytokine Expression in Peri-Burn Skin

**Figure 5:**
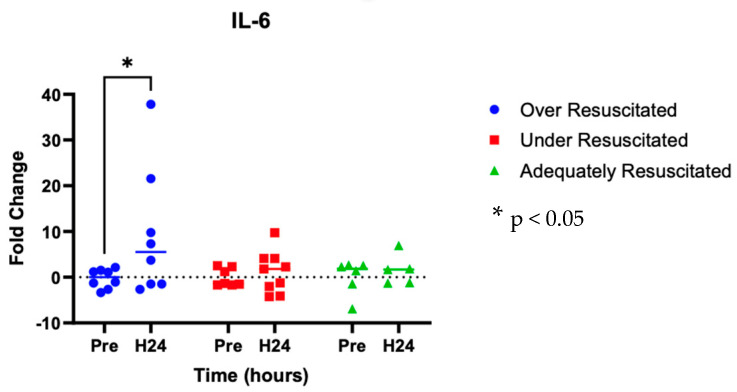
Cytokine analysis demonstrated increased expression of IL-6 to the peri-burn site of over-resuscitated animals at H24.

**Figure 6:**
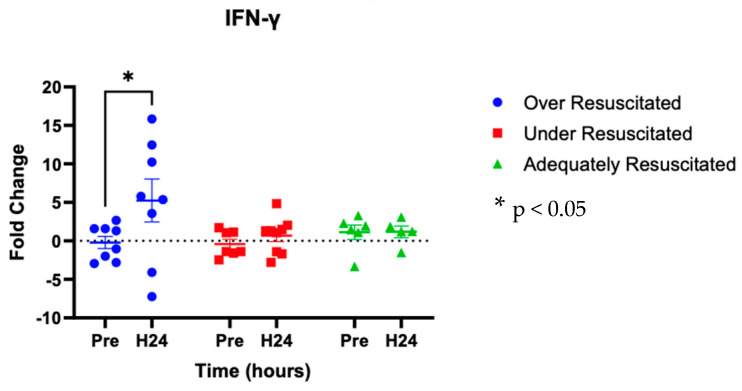
Cytokine analysis demonstrated increased expression of IL-y to the peri-burn site of over-resuscitated animals at H24.

**Figure 7:**
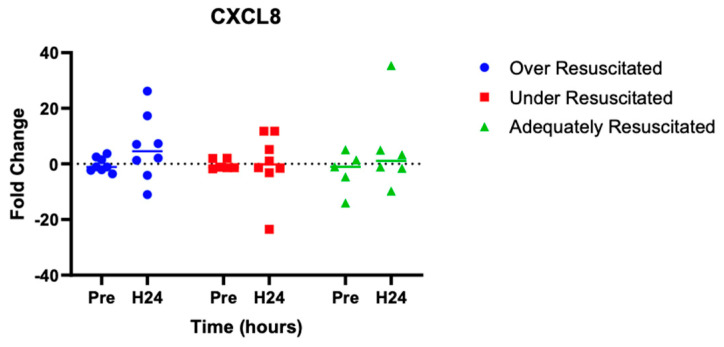
Cytokine analysis demonstrated no significant expression of CXCL8 to the peri-burn site of over-resuscitated animals at H24.

At hour 24, there was an increased expression of two cytokines in over-resuscitated animals in the peri-burn skin compared to pre-injury skin (IL-6: *p* = 0.0220, IFN-y: *p* = 0.0253, respectively) (Figure 5 and Figure 6). At hour 24, there was no differential expression of CXCL8 in peri-burn skin compared to pre-injury skin (OR: *p* = 0.5281, AR: *p* = 0.4754, UR: >0.9999) (Figure 7). Under- and adequately resuscitated animals did not have any differential expression of the examined cytokines at hour 24 compared to uninjured animals. All values are represented as fold changes.

### 3.4. Resuscitation Levels Affect Expression of TUNEL

**Figure 8:**
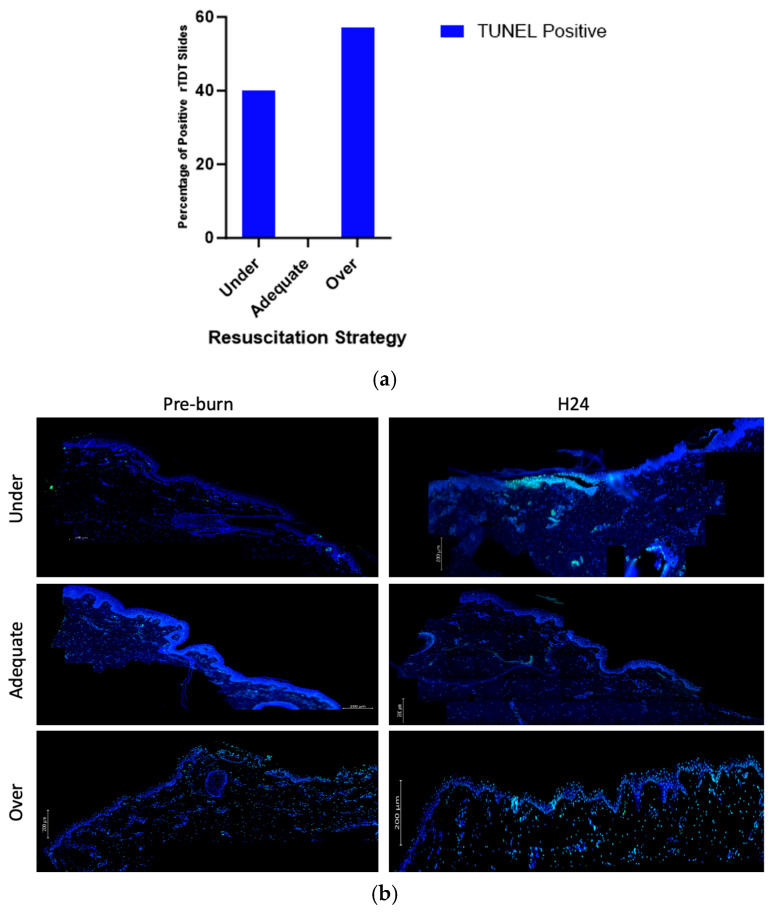
(**a**) Contingency of TUNEL positivity with regards to fluid resuscitation shows over-resuscitated animals expressed more % rTDT positivity compared to other groups. (**b**) Representative images displaying abundance of rTDT across hours pre-burn and at hour 24 by resuscitation group.

Compared to other groups, over- and under-resuscitated animals showed more % positive rTDT staining compared to the adequately and under-resuscitated groups in peri-burn skin at H24 (Figure 8a). Representative images displaying the abundance of rTDT can be seen in Figure 8b.

## 4. Discussion

Conventionally, a majority of the research studies on severe burn resuscitation have been conducted at the systemic level and less in the local tissue environment. Further, research has traditionally focused on the zone of coagulation, or tissue that rapidly becomes necrotic following burn injury. While necessary, it has become more apparent in recent years that more research is needed to examine the dynamic local environment directly surrounding the zone of coagulation, also known as the zone of stasis. That said, ex vivo models of skin poised to study the local environment are limited by the fact that the local environment is highly influenced by systemic factors such as plasma circulating cytokines [20]. Conversely, in vivo limitations to studying burn progression are due to inconsistencies with burn depth, severity, and location, resulting in constraints such as the inability to accurately control fluid resuscitation levels. By leveraging an experimental model of swine, this study examined the local burn environment in the context of more prominent systemic factors like ischemia. Further, by manipulating levels of fluid resuscitation in swine in burn shock, we examined the impact of fluid on the cells within the zone of stasis.

The pathophysiology behind the proinflammatory cascade of events that subsequently occurs systemically following thermal injury is known to contribute to the transition from partial thickness to deep partial or full-thickness burns [21]. The proliferation of cytokines such as TNF-α, INF-γ, IL-6, and IL-8 have been identified in humans but are typically dependent on wound size and the presence of sepsis [22,23,24]. Further, details pertinent to specific differences in innate versus adaptive immunity following thermal injury have been raised and have been previously described by Keyloun et al. and Korkmaz et al. [21,23].

Examining specifically the peri-burn skin, this study found that over-resuscitated animals at hour 2 following injury had significantly increased perfusion to the zone of stasis compared to under-resuscitated animals. While increased perfusion to the zone of stasis limits capillary vasoconstriction and ischemia, it is still critical to consider the implications known to stem from over-resuscitation in patients suffering from large thermal burns. In 2000, Dr. Basil Prutt coined the term “fluid creep” to describe complications (i.e., compartment syndrome, infections, ARDS) that arise due to over-resuscitation. This paper has detailed an additional complication that may arrive from over-resuscitation: the exacerbation of burn wound progression.

Concurrent with increased perfusion to peri-burn skin, this study also found a significantly elevated gene expression of cytokines such as interleukin-6 (IL-6) in the zone of stasis at hour 24 following injury. The role of IL-6 in thermal burns is multifaceted. Systemically, in the hours and days following burn injury, IL-6 leads to the synthesis of acute phase protein in the liver and induces the differentiation of naïve T cells [23]. Locally, IL-6 is thought to be transcribed by epidermal keratinocytes following increased vasodilation. That said, in the hours, days, and even months following burn injury, IL-6 has also been found to be significantly proportional to burn size and depth [23]. This finding is consistent with this paper’s hypothesis that as the zone of stasis expands, levels of IL-6 will increase significantly in over-resuscitated animals.

Also expected is our finding of increased expression of IFN-γ at hour 24 following burn injury. As another crucial element of the innate immune system, IFN-γ has previously been shown to be elevated in the blood of burn patients, but not significantly correlated to burn size. Released by natural killer cells, macrophages, and antigen-presenting cells, IFN-γ inhibits collagen synthesis [19]. An abundance of IFN-γ has therefore, in turn, been shown to slow healing [25]. The finding that IFN-γ is significantly increased in over-resuscitated animals at hour 24 supports the idea that burn wound progression in these animals inhibits mechanisms behind wound healing in the zone of stasis. Taken together with our perfusion findings, our results that both IL-6 and IFN-y are increased at hour 24 can also be contextualized vascularly. Severe burns can increase vascular permeability by adversely influencing several factors that contribute to the structural integrity of the intravascular lumen. For example, reactive oxygen species, nitrogen substances, matrix metalloproteinases, etc. have all been found to cause the destruction of the glycocalyx [26]. As a result, cytokines such as IL-6 and IFN-y can leak out of the vasculature, infringing on the dynamic local environment.

The release of CXCL8 is important for the recruitment of neutrophils, a type of white blood cell produced in the bone marrow, to the wound site, where it targets pathogens and releases additional cytokines. Like IL-6, it has been found to rise significantly in the hours, days, and weeks following a major burn injury [23,27]. Thus, the insignificance of IL-8 between pre-burn and H24, as well as between resuscitation groups, was unexpected in this study.

While we have discussed possible mechanisms behind why IL-6 and IFN-y were elevated in the peri-burn space following severe burn injury, the exact mechanism behind why early excessive fluid resuscitation specifically leads to significant increases in the immunological mechanisms behind burn wound progression (e.g., elevated IL-6 and IFN-γ) is less understood in the literature. However, the results of this study form a hypothesis that the complications of fluid creep such as compartment syndrome can directly lead to increases in inflammatory cytokines like IL-6 and IFN-γ in the zone of stasis. This could result due to rises in intercompartmental pressure, which, in turn, could damage the microcirculation of the zone of stasis, leading to tissue anoxia and cell death—conditions tied to enhanced T-cell activation [28].

Supporting our immunological and perfusion data is our finding that the percent positivity of rTdT was, on average, elevated in over-resuscitated animals compared to under- and adequately resuscitated animals. As a means to visualize the presence of DNA strand breaks, rTdT has historically been a reliable measure of apoptosis [29,30]. Here, it was analyzed as a potential functional outcome of the cytokine expression that was visualized in the peri-burn skin. While not conclusive, there was more TUNEL expression in the over- and under-resuscitated groups compared to the adequately resuscitated group, suggesting a role for apoptosis in burn conversion in the peri-burn skin.

Ultimately, the immunological and perfusion data suggest that one way to limit burn wound progression would be to provide adequate fluid resuscitation levels without under- or over-resuscitating. Adequate resuscitation, therefore, is not only critical for preventing internal organ dysfunction but also for ensuring that necrotic tissue does not spread and lead to downstream comorbidities. Furthermore, therapeutics designed to reduce the production of proinflammatory cytokines directly following burn injury should also be explored given the proximity of the zone of stasis to necrotic tissue and the limited amount of time available to limit the spread of progression. Designing therapeutics that target the specific molecular underpinnings driving burn progression have the potential to reduce morbidity, mortality, and the psychological and long-lasting physical implications of large burns [31].

Recent work has already begun exploring how to reduce inflammation-driven burn conversion. In 2021, Dolgachev et al. published findings indicating that in swine, an oil-in-water nano emulsion formulation (NB-201) containing benzalkonium chloride prevented burn progression by the downregulation of neutrophils [32]. Additional research in both swine and rodent full-thickness burn wounds has found that metal chelation acts to reduce burn wound progression through the reduction in the expression of IL-6 [33]. Further, active research is investigating the use of mesenchymal stem cells, biomaterials, and immune regulators as a means to reduce cell death, improve wound reperfusion, and promote tissue regrowth [12].

Future research should continue to pursue ways to mitigate burn wound progression via preventative measures, the design of novel therapeutics, and the invention of additional metrics to quantify adequate fluid resuscitation.

There exist several relevant limitations that warrant consideration. Firstly, we note the confounding effect of repeated biopsy sampling. Each biopsy created an acute wound, which itself could have possibly influenced local tissue inflammatory dynamics and perfusion. Further, while tissue biopsies were performed in a standardized fashion across regions deemed to represent peri-burn skin, it was not possible to confirm with absolute certainty that the sampled areas contained exclusively peri-burn tissue. Localization was based on gross inspection guided by LDI. However, LDI has known limitations, including motion artifact. Another limitation is that adjacent LDI values that fall just above or below preset thresholds may be visually interpreted as distinct injury zones despite representing minimal actual differences [34]. As such, some biopsies may have inadvertently included partially injured or nonviable skin, which could have influenced the downstream analyses.

An additional limitation of this study stemmed from our finding that both the under- and adequately resuscitated animal groups recorded urine outputs at 1.12 ± 0.16 (under resuscitated) and 1.82 ± 0.27 mL/kg/h (adequately resuscitated), respectfully (Figure 3b), which were not significant from one another. This could mean that tissues could potentially have been adequately perfused for both groups, with implications for downstream immunological and perfusion effects. This study also investigated three cytokines very relevant to burn immunology but did not include an analysis of additional cytokines that shared equal relevance such as TNF-α. Future research examining local cytokine expression in the zone of stasis should include profiles of additional immunological markers such as TNF-α. Lastly, a significant number of LDI assessments were excluded from analysis due to poor image quality, including issues such as motion artifact or low resolution, which limited the completeness of perfusion data for the over-resuscitated animal group at hours 12 and 24.

## 5. Conclusions

Based on the resuscitation strategy, this animal model showed differences in perfusion and inflammatory cytokine expression in peri-burn skin samples. Varying levels of resuscitation following a burn can have wide-ranging consequences that may affect burn wound progression.

Under- or over-resuscitation may lead to local changes in the burn wound that could affect the overall severity and evolution of the injury over time. Judicious fluid resuscitation may not only be useful in dampening the possible systemic complications associated with fluid creep (e.g., compartment syndrome) but may have direct effects on the local wound bed. By further elucidating mechanisms for burn conversion, interventions may be developed.

## Data Availability

The data presented in this study are available on request from the corresponding author.

## Abbreviations

The following abbreviations have been used in this manuscript:

TBSA	Total body surface area
LDI	Laser Doppler Imaging
RNA	Ribonucleic acid
qRT-PCR	Quantitative reverse transcription–polymerase chain reaction
IL-6	Interleukin 6
CXCL8	Chemokine (C-X-C motif) ligand 8
IFN-γ	Interferon-gamma
TUNEL	Terminal deoxynucleotidyl-transferase-mediated dUTP nick-end labeling
BWP	Burn wound progression
IV	Intravenous
LR	Lactated ringer
UO	Urine output
ROS	Reactive oxygen species
AAALAC	Association for Assessment and Accreditation of Laboratory Animal Care International
PHS	Public Health Service
IACUC	Institutional Animal Care and Use Committee
AR	Adequate resuscitation
UR	Under-resuscitation
OR	Over-resuscitation
ANOVA	Analysis of variance
rTDT	Recombinant terminal deoxynucleotidyl transferase
DAPI	4′,6-Diamidino-2-phenylindole
DI	H_2_O de-ionized water
SCC	Saline-sodium citrate
GFP	Green fluorescent protein
ARDS	Acute respiratory distress syndrome
TNF-α	Tumor necrosis factor-alpha
